# Diversity of SIRV-like Viruses from a North American Population

**DOI:** 10.3390/v14071439

**Published:** 2022-06-30

**Authors:** Joseph R. Fackler, Michael Dworjan, Khaled S. Gazi, Dennis W. Grogan

**Affiliations:** 1 Department of Biological Sciences, University of Cincinnati, Cincinnati, OH 45221-0006, USA; jfackler@aphage.com (J.R.F.); msw2109@caa.columbia.edu (M.D.); kgazi@bu.edu.sa (K.S.G.); 2 Adaptive Phage Therapeutics, Gaithersburg, MD 20878, USA; 3 Department of Biology, Faculty of Science and Arts in Almandaq, Al-Baha University, Almandaq 65756, Saudi Arabia

**Keywords:** Sulfolobus islandicus rod-shaped virus (SIRV), archaeal viruses, Sulfolobales, local populations, resistant host variants, virion stability, natural variation

## Abstract

A small subset of acidic hot springs sampled in Yellowstone National Park yielded rod-shaped viruses which lysed liquid host cultures and formed clear plaques on lawns of host cells. Three isolates chosen for detailed analysis were found to be genetically related to previously described isolates of the Sulfolobus islandicus rod-shaped virus (SIRV), but distinct from them and from each other. Functional stability of the new isolates was assessed in a series of inactivation experiments. UV-C radiation inactivated one of the isolates somewhat faster than bacteriophage λ, suggesting that encapsidation in the SIRV-like virion did not confer unusual protection of the DNA from UV damage. With respect to high temperature, the new isolates were extremely, but not equally, stable. Several chemical treatments were found to inactivate the virions and, in some cases, to reveal apparent differences in virion stability among the isolates. Screening a larger set of isolates identified greater variation of these stability properties but found few correlations among the resulting profiles. The majority of host cells infected by the new isolates were killed, but survivors exhibited heritable resistance, which could not be attributed to CRISPR spacer acquisition or the loss of the pilus-related genes identified by earlier studies. Virus-resistant host variants arose at high frequency and most were resistant to multiple viral strains; conversely, resistant host clones generated virus-sensitive variants, also at high frequency. Virus-resistant cells lacked the ability of virus-sensitive cells to bind virions in liquid suspensions. Rapid interconversion of sensitive and resistant forms of a host strain suggests the operation of a yet-unidentified mechanism that acts to allow both the lytic virus and its host to propagate in highly localized natural populations, whereas variation of virion-stability phenotypes among the new viral isolates suggests that multiple molecular features contribute to the biological durability of these viruses.

## 1. Introduction

Viruses that infect unicellular organisms play crucial roles in (i) global ecology, by controlling microbial population sizes, (ii) microbial evolution, by promoting diversification and genetic transfer, and (iii) microbiological research, by providing experimental tools for analysis and manipulation of host cells at the molecular level. The deep evolutionary divergence of archaea revealed by molecular phylogeny [1] predicts a corresponding diversity of archaeal viruses. Although some archaeal viruses can be grouped into well-established families that include viruses of eukaryotes or bacteria [2,3], many define new families known only to infect archaea [4,5,6]. Viruses of the archaea that populate geothermal habitats are particularly diverse, and some exhibit functional or morphological features that have no precedent among eukaryotic or bacterial viruses. These unique features include extracellular maturation of virions [7], virion release through virus-encoded protein structures in the host cell membrane [8,9], and encapsidation of dsDNA in the A conformation [10].

Thermoacidophilic archaea of the order Sulfolobales, which include the recognized genera *Sulfolobus* and *Saccharolobus* [11] and numerous *Saccharolobus*-related isolates informally designated “*Sulfolobus islandicus*” [12], offer practical advantages for systematic study, reflecting their comparatively accessible habitat and facile cultivation relative to many other hyperthermophilic archaea. These archaea also serve as hosts for several viruses, including the Sulfolobus islandicus rod-shaped virus (SIRV), which have been isolated from acidic hot springs in Iceland and North America [13,14,15]. The relatively simple structure of the SIRV virion represents a helix formed by tight binding of a 134-aa major capsid protein to the linear dsDNA genome and self-association of the DNA-bound subunits [10]. Both the major capsid protein and a larger, less abundant capsid protein have been found to be glycosylated [16]. To the best of our knowledge, however, neither the functional stability of SIRV virions nor natural variation in stability properties have been documented.

In this study we isolated genetically diverse SIRV-like viruses from a previously unreported local population in Yellowstone National Park (YNP). Plaque formation by these isolates facilitated measurement of virion stability under various experimental conditions. The results indicate that the virions are extremely stable, although rates of inactivation varied significantly among different treatments and different viral isolates. Our results also indicate that a host strain can interconvert between virus-sensitive and virus-resistant forms. The observed resistance reflects failure of host cells to adsorb infective virions, but this property could not be attributed to mutation of known pilus-related genes.

## 2. Materials and Methods

### 2.1. Strains and Growth Conditions

Environmental samples were collected from thermal areas in YNP (WY, USA) in 2008, 2011, and 2014 under research permit YELL-05382. Hot-spring fluid samples were sealed in capped glass vials, transported and stored at ambient temperature, and plated for direct isolation within 7–10 d of collection as previously described [17,18,19]. Samples that yielded plaque-forming units (PFU) came from the drainage of a 2 m × 3 m acidic pool located south of Rabbit Creek in the Midway Geyser Basin, or from pools in the Ragged Hills section of Norris Geyser Basin (Table 1).

Potential host strains screened for virus sensitivity were isolated from similar environmental samples collected in 1999 or 2000 from various regions of YNP, and by other researchers from the Pisciarelli solfatara (near Pozzuoli, Italy), the Kamchatka peninsula of eastern Russia, and Lassen Volcanic National Park in northern California, USA. Unless otherwise noted, the host strain for virus propagation was Kamchatka isolate K00 16-4 [17], which has also been designated M16.4 [19]. Host cultures were grown on dextrin-tryptone (DT) medium and incubated aerobically at 78–80 °C, as previously described [20]. Liquid cultures were grown to a density of about 5 × 10^7^ cells per mL (typically 15 to 36 h incubation) before infection. After infection, cultures were incubated until turbidity decreased by at least half and remained stable over time (typically 15 to 48 h). The resulting lysates were purified of cells and particulate debris by centrifugation (11,000× *g* 15 min) and the supernatants were stored at 4 °C. Overlays for plaque formation were produced by pouring 4 mL of 0.3% Gellan gum, 0.01% glutamic acid containing approximately 5 × 10^7^ host cells over the surface of a plate and allowing about 15 min for the overlay to harden before incubation. Overlay plates were incubated 3 d for plaque formation, unless otherwise noted. Viable cell counts of environmental samples were determined by spreading liquid/sediment mixtures over the surface of plates and counting colonies after 7–10 d incubation [17,18].

### 2.2. DNA Analyses

To purify viral DNA, liquid lysates (10^8^ to 10^9^ PFU/mL) were clarified by centrifugation, and virions in the supernatant were precipitated by NaCl and PEG 8000, as described in [13]. The resulting precipitate was collected by centrifugation and extracted either by phenol-chloroform or by a guanidinium thiocyanate/diatomaceous earth mixture [21], followed in either case by ethanol precipitation. Purified DNAs were sequenced by the Purdue University Genomics Core Facility (West Lafayette, Indiana) by preparation of a plasmid library via shotgun cloning in *E. coli*, sequencing of inserts, and assembly into contigs (in the case of the V65 genome, one gap was closed by PCR). Unless otherwise noted, PCR analyses in the present study used the primers listed in Table S1 and the following program: (1) 95°, 2 min; (2) 95°, 22 s; (3) 50°, 22 s; (4) 72°, 90 s; (5) repeat (2–4) 30 times; (6) 72°, 3 min.

Viral genome sequences were aligned with progressive MAUVE [22] in Geneious Prime (version 2022.0) using default parameters. Collinear blocks were concatenated and the resulting alignments used to infer neighbor-joining trees using a Tamura-Nei model, with resampling and a nodal threshold of 70% support, using Geneious-Prime. ORFs represented in the V3, V60, V65, SIRV1 and SIRV2 genomes and encoding proteins of more than 80 amino acids were aligned using Clustal W. Non-synonymous base substitutions per non-synonymous site (Ka) and synonymous base substitutions per synonymous site (Ks) were tabulated from each alignment using the Synonymous Non-synonymous Analysis Program (SNAP, http://www.hiv.lanl.gov (accessed on 30 May 2015)).

### 2.3. Electron Microscopy

Virion suspensions were adsorbed onto glow-discharged, formvar-coated grids and stained with 2% uranyl acetate. Grids were imaged in a JEOL JEM 2100 TEM, operated at 200 kV with a LaB6 filament. Digital micrographs were captured with a Gatan 833 side-mounted camera.

### 2.4. Inactivation Assays

To measure thermal inactivation, virion suspensions, diluted to titers of 10^3^ to 10^5^ PFU per mL in *Sulfolobus* dilution buffer (S_dil_) [23], were sealed in polypropylene tubes and incubated at the test temperature for a defined length of time (usually 1 h). Surviving PFU were enumerated by plaque assay and normalized by the titer of a corresponding suspension held at room temperature. To measure inactivation by detergents or chaotropes, concentrated stock solutions of the additive were adjusted to a pH of 3–5 with dilute H_2_SO_4_, and were added to an equal volume of virion suspensions in S_dil_ at a titer of about 10^8^ PFU per mL. The mixture was incubated one hour at 70 °C, cooled to room temperature, and diluted 100-fold in S_dil_ to stop inactivation. Aliquots (50 μL) of 10-, 100-, and 1000-fold dilutions were then plated as described above. To measure the effects of chloroform or protease, the virion suspensions were buffered by 0.25 M (each) potassium phosphate and malate (pH 6.0), and were incubated with the test reagent for one hour, followed by dilution in S_dil_ and plaque assay. The proteases tested were trypsin, Proteinase K, and Pronase, all at a concentration of 10 mg per mL. Assays with sodium periodate used sodium phosphate (pH 6.0) as the pH buffer and were chemically quenched by adding 1 M sucrose before dilution and plating.

To measure the effect of pH, 100 μL virion suspensions in 0.1X S_dil_ were added to 10 μL of concentrated pH buffer, yielding a final concentration of 90 mM of the buffering species. The resulting mixtures were incubated 1 h at 70 °C, then were diluted 10-fold into the S_dil_ and further diluted for counting by plaque assay. Most intervals of the pH range were tested independently by two different buffering mixtures; unless otherwise noted, the counter-ions added during pH adjustment were potassium or sulfate. The buffering mixtures used at the indicated pH ranges were: H_2_SO_4_ (for pH ≤ 2), equimolar citric and phosphoric acids (for pH 2–4), equimolar MES and TAPS (for pH 5–9), equimolar malic and phosphoric acids (for pH 3–4.5), diethanolamine titrated with phosphoric acid (for pH 9–10.5), and potassium phosphate (for pH > 11).

UV irradiation was performed on 5 mL suspensions agitated in disposable 9 cm Petri dishes under a 25 W germicidal lamp and plated under dim red light; UV-C intensity was measured by a calibrated windowless radiometer (International Light, Inc., Peabody, MA, USA). Bacteriophage λ suspensions were plated on *E. coli* strain MC4100 using tryptone agar overlays (0.7%) on 1% tryptone agar plates.

### 2.5. Measurements of Acquired Resistance

For fluctuation analysis, isolated colonies of K00 16-4 were picked while still small and grown in 1 mL liquid DT medium overnight to produce 20 independent cultures of 1–2 × 10^8^ cells each. To facilitate scoring, each culture was supplemented with 9% DMSO, dispensed into equal aliquots of 0.1 mL, and stored frozen at −70 °C. Aliquots were analyzed by thawing and serial dilution (10^−1^ to 10^−6^). To enumerate total viable cells, the 10^−4^, 10^−5^, and 10^−6^ dilutions were spread on solid DT media and incubated. To enumerate virus-resistant cells, 180 μL of the 10^−3^ dilution (approximately 2 × 10^4^ CFU) was pipetted into a separate microtube to which 20 µL of lysate, corresponding to 5–20 × 10^7^ PFU, was added. Each tube was sealed and incubated for 1 h at 80 °C to allow for virion adsorption, and then the entire mixture was spread on the surface of a plate and incubated for colony formation. To confirm that the clarified lysate contained no viable cells, 200 μL of each lysate was spread on a separate DT plate and incubated. The average number of cells per culture and the number of resistant cells in each independent culture were determined from the resulting colony counts, and recorded and analyzed via the FALCOR web interface [24]. In addition, virus-resistant colonies identified in the analysis were streaked for isolation. Isolated colonies were re-streaked on DT plates, and resulting colonies were re-suspended in 9% DMSO solution and cross-streaked against virus on plates to confirm the resistance phenotype (see below). Phenotypically confirmed cell suspensions were cryo-preserved at −70 °C.

To score virus resistance, clarified lysate diluted 1:2 with S_dil_ (400 µL) was spread down the center of a DT plate (~3 cm in width). After absorption of the liquid, colonies of host strains were touched with a sterile applicator and drawn across the plate and through the lysate zone.

To detect non-selected resistance, a single colony of sensitive host K00 16-4 was grown to a density of 9 × 10^8^ cells/mL in a volume of 4 mL liquid DT medium, and aliquots of a dilution series were spread plated on DT plates to generate isolated colonies. Individual colonies were transferred to corresponding aliquots (100 µL) of S_dil_ buffer in a microtiter plate, and 5 µL of each suspension was replica-plated by pipetting onto four DT plates spread with 100 µL of S_dil_ (control) or lysate of V3, V60, or V65. After a 4 day incubation, resistance was scored by comparing growth on SIRV plates to that of the control plate. Colonies scored as resistant were re-streaked and stored in 9% DMSO at −80 °C for further analysis. Detection and recovery of SIRV-sensitive clones followed the same procedure, except that the initial culture was grown from a V3-, V60-, or V65-resistant clone to a density of 1.4 × 10^8^ cells/mL, and those isolated colonies scored as sensitive were retained for analysis.

### 2.6. Virion Binding Assays

Cells to be evaluated were grown in liquid DT medium from an isolated colony, washed in S_dil_ buffer, and resuspended to a density of 1.6 × 10^9^ CFU per mL, as confirmed by plating. To 25 µL of the resulting cell suspension in a PCR tube, 1.0 µL of virus suspension (1.7 × 10^5^ PFU) was added, yielding a multiplicity of infection (MOI) of 4 × 10^−3^; virions added to a corresponding 25 µL of S_dil_ buffer provided a no-cell control for each strain being evaluated. Immediately after mixing, the suspensions were incubated at 70 °C; samples (2.5 µL) were withdrawn after 2, 15, and 75 min incubation and were diluted immediately into 1.00 mL room-temperature S_dil_ in a microcentrifuge tube. This diluted but unfractionated mixture was sampled for PFU (as described below) and then centrifuged 10 min at 10,000× *g*; the resulting supernatant was sampled for PFU. The rest of the supernatant was discarded by careful decanting, and the remaining pellet fraction (including about 10 µL liquid) was washed by resuspension in 0.5 mL S_dil_ and repetition of the centrifugation and decanting steps. Finally, the resulting washed pellet fraction was resuspended in 1.00 mL S_dil_ and sampled for PFU. In each case (i.e., the unfractionated mixture, supernatant, and pellet fraction of each time point), a 25 µL sample was transferred to an overlay medium containing *S. islandicus* strain K00 16-4 and plated on solid DT medium; plaques were counted after 3 d of incubation. To assess binding, the numbers of PFU in all sampled fractions were normalized to the number of PFU added to the cell suspension.

## 3. Results

### 3.1. New Isolates of Lytic Archaeal Virus

**Figure 1:**
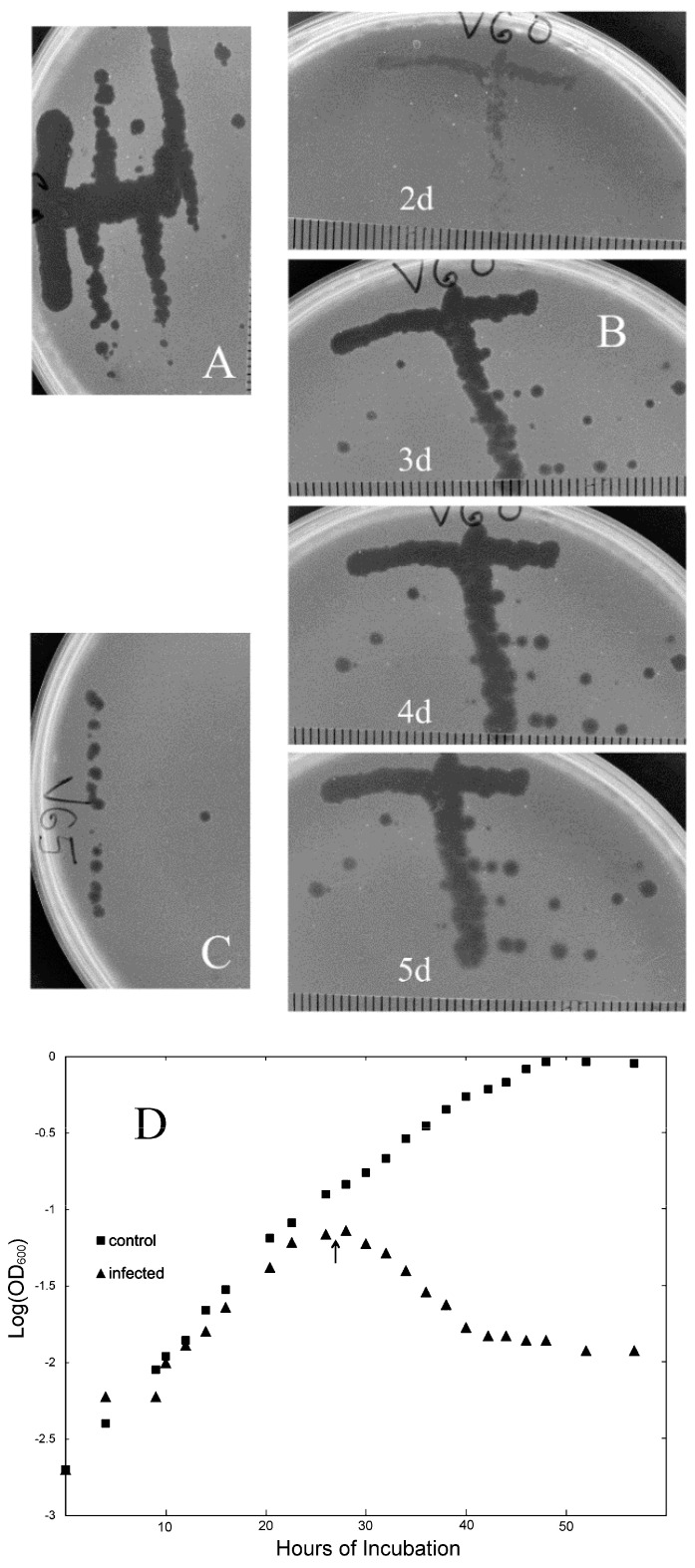
Lytic properties of viral isolates. Panels (**A**–**C**): Plaque formation. Suspensions of virions were streaked over the surface of overlays containing cells of strain K00 16-4. All photographs have the same scale, as indicated by the mm ruling. Panel (**A**): V3 at 3 days incubation; panel (**B**): V60 at the indicated days of incubation; panel (**C**): V65 at 3 days incubation; panel (**D**): infection in liquid. A growing culture of host strain K00 16-4 was diluted into fresh dextrin-tryptone medium to yield 2 × 10^8^ cells in a total volume of 88 mL; this mixture was then divided equally between two 250 mL Erlenmeyer flasks. The resulting duplicate cultures were aerated by swirling at 150 rpm, and 1 mL aliquots were withdrawn at the times indicated. Turbidity was measured in 1 cm cuvettes and samples were diluted by a defined ratio with S_dil_ when necessary to ensure proportionality between turbidity and cell density (i.e., OD_600_ < 0.2). To start the infection, 3 × 10^9^ PFU of isolate V60 was added at the time indicated by the arrows, yielding a calculated MOI of 7.

In the course of a culturing survey of YNP acidic geothermal sites in 2008, we observed that samples drawn from a particular geothermal system (which we designate “SRC”), located in the Midway Geyser Basin south of Rabbit Creek, produced nearly confluent colonies when spread directly on plates (i.e., without enrichment) and that some of these plates also exhibited small circular areas in which colonial growth was inhibited (i.e., lacunae). To investigate the nature of this inhibition, the original samples were re-plated in overlays containing various *S. islandicus* strains clonally purified from the same and nearby environmental samples, included as potential hosts. This initial plating resulted in several cases of plaque formation. In most cases, infectivity of the plaque-forming agent was confirmed by picking and spotting or streaking onto fresh overlays of the same or different host strains. After clonal purification by streaking (illustrated in Figure 1A–C), 13 of the plaque-forming agents were characterized. Three of them, designated V3, V60, and V65, exhibited reliable lytic propagation under various laboratory conditions yet differed with respect to genomic restriction patterns, and therefore were chosen for more extensive analysis.

**Figure 2:**
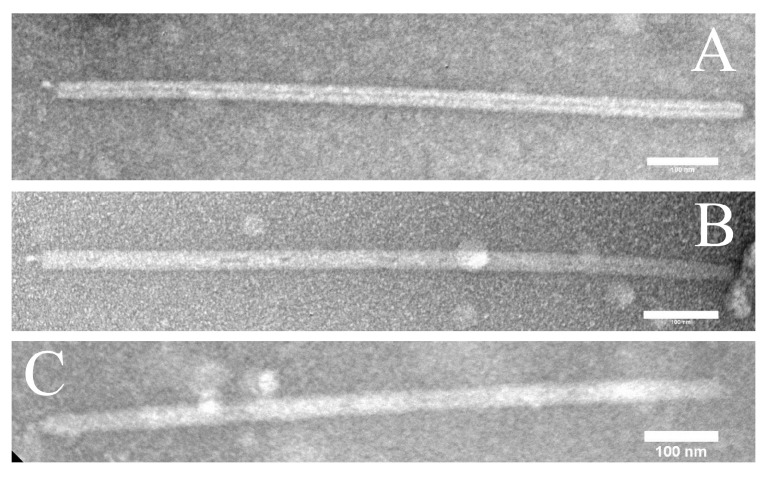
Virion Morphology. Transmission EM of lysates stained with uranyl acetate (see Section 2). Panel (**A**): isolate V3; panel (**B**): isolate V60; panel (**C**): isolate V65.

In overlay plates, initially turbid plaques became transparent and continued to enlarge as incubation continued (Figure 1A–C). In liquid cultures, exponential growth stopped soon after infection and turbidity declined steadily to low levels (Figure 1D). Lysates typically had visible stringy or clumped debris, with titers ranging from 10^7^ to 10^9^ PFU per mL. Thus, the viruses we examined had impacts on *S. islandicus* cultures similar to those of lytic bacteriophages on their bacterial hosts. EM of negatively stained lysates of the three isolates (see Section 2) revealed large numbers of straight, rod-shaped particles 900–950 nm long and approximately 28 nm in diameter (Figure 2A–C). For all three isolates, virion precipitation, DNA extraction, and restriction endonuclease digestion yielded various patterns of DNA fragments, the lengths of which totaled 35–36 kbp. In addition, pairs of viral restriction fragments were observed to survive heat-denaturation and rapid cooling, which was not observed for any restriction fragments of control DNA (Figure S1 lanes B vs. D).

**Figure 3:**
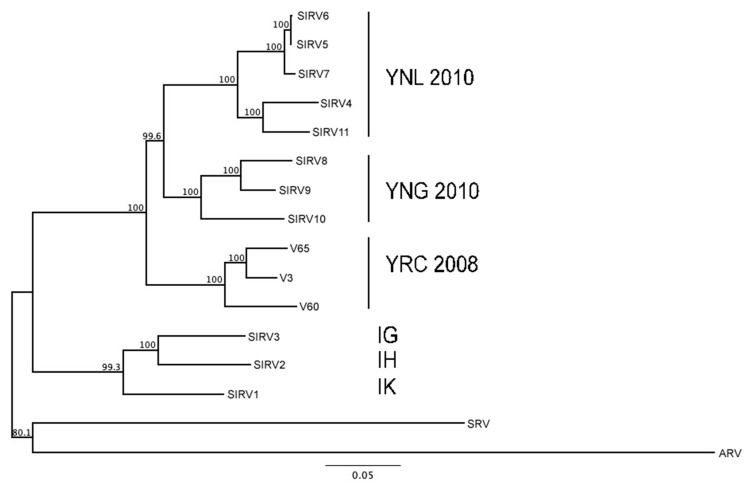
Relatedness of viral isolates. Genome sequences of the indicated viruses were analyzed by progressive MAUVE (see Section 2). Abbreviations: ARV, Acidianus Rod-shaped Virus 1 (NC_009965.1); SRV, Stygiolobus Rod-shaped Virus (NC_025375.1). GenBank accession numbers for SIRV1–11 are NC_004087.1, NC_004086.1, NC_030884, KY744231, KY744233, KY744235, KY744232, KY744229, KY744228, KY744230, and KY744234, respectively. Geographical regions are indicated as follows: YNL, Yellowstone National Park (Nymph Lake); YNG, Yellowstone National Park (Norris Geyser Basin); YRC, Yellowstone National Park (Rabbit Creek); IG, Iceland (Gunnuhver); IH, Iceland (Hveragerdi); IK, Iceland (Kverkjoll). If known, the year of isolation is also shown with location; for additional details, see Table 1.

This combination of observed properties, i.e., infection of *S. islandicus* strains, cell lysis, virion morphology, and length and rapid renaturation of viral DNA, had previously been reported for SIRV1 and SIRV2 [8,12,13,15]. Sequencing of V3, V60, and V65 DNAs confirmed that each is genetically unique but related to the other two isolates and to 11 other SIRV-like isolates from YNP that have been reported previously (Table 1; Figure 3). We also noted that, within this set of viruses, the pattern of genome similarity correlated with geographical origin, as the smallest clades represented the spatially closest isolates, whereas the deepest divergence separated the Icelandic isolates from the North American isolates (Figure 3 and Table 1). More detailed analysis of genome sequences revealed overall similarity of genetic organization among SIRV1, SIRV2, and the three new isolates (Figure S2), as well as divergence separating all North American isolates from all Icelandic isolates. This pattern is consistent with the dendrogram of Figure 3 and analyses of other SIRV-like viruses isolated from YNP [15].

Properties of individual genes conserved among these five genomes are listed in Table S2. The dN/dS ratio (Ka/Ks; non-synonymous to synonymous substitutions) provides a criterion for detecting either negative (purifying) selection or positive (diversifying) selection on a protein, and thus potential evidence regarding function. The dN/dS values for ORFs represented in all five genomes ranged from 0.083 to 1.79 (Table S2), suggesting significant differences in the nature and intensity of selective pressure. Most of the ORFs found in all five SIRVs yielded dN/dS < 1, similar to chromosomal genes of the host species [25]. Of the shared ORFs, the one exhibiting the most stringent constraint on amino acid sequence was the 134-amino-acid major capsid protein. Among all five genomes, it exhibited an extremely low dN/dS ratio (0.083), whereas among the three new isolates, its amino acid sequence did not vary, despite base-pair substitutions at 35 positions within the coding region (Figure S3). This high degree of amino acid conservation could not be attributed to the short length or essential role of the polypeptide, as all the shorter ORFs included in the analysis yielded markedly higher dN/dS values and also are expected to be essential for SIRV propagation. Specifically, homologs of SIRV1 gp9 and gp27 are implicated in genome replication, whereas the homologs of SIRV1 gp42 control the process of opening the host cell envelope to release mature virions (Table S2). The unusually high conservation of the major capsid protein’s primary structure versus these other small proteins thus indicates the critical importance of the multiple, specific subunit-subunit and subunit-DNA interactions required by its functional role as the primary structural element of SIRV virions.

In contrast, another capsid protein was found to represent one of the most diversified genes in the five viral genomes. Specifically, the much larger and less abundant capsid protein represented by SIRV1 gp30 (Table S2) has one of the highest dN/dS ratios of those analyzed. Previous studies have indicated that this protein forms the filaments seen at the ends of SIRV virions and mediates one or more early steps of infection [26]. Since the specificity of virion binding to the cell surface often provides the host with the opportunity to avoid infection by altering the relevant receptor protein(s), the viral protein involved in binding typically is under selection for adapting to this altered amino acid sequence or to an alternative host protein [27]. The high divergence observed for this capsid protein may, accordingly, reflect an analogous process.

### 3.2. Virion Stability

Understanding how biological function can be maintained under severe environmental conditions has motivated much of the research on hyperthermophilic archaea but has not been a focus in studies of their viruses. SIRV particles have a relatively simple structure and composition dominated by the small capsid protein, which binds viral DNA, holds it in the A conformation, and self-associates to form the compact rod-shaped helix characteristic of Rudivirus morphology [10,16]. At least two of the virion proteins (the small major capsid protein and a larger, less abundant capsid protein) are glycosylated [16]. These two virion features, i.e., the simple, compact structure and the glycosylation of capsid proteins, seem well suited to preserve native structure and infectivity in a hot and acidic environment, but this stability had not been examined in biological terms. We therefore took advantage of the quantitative assays provided by plaque formation to evaluate the biological stability of the viral isolates toward agents with well-characterized effects on nucleic acids or proteins.

**Figure 4:**
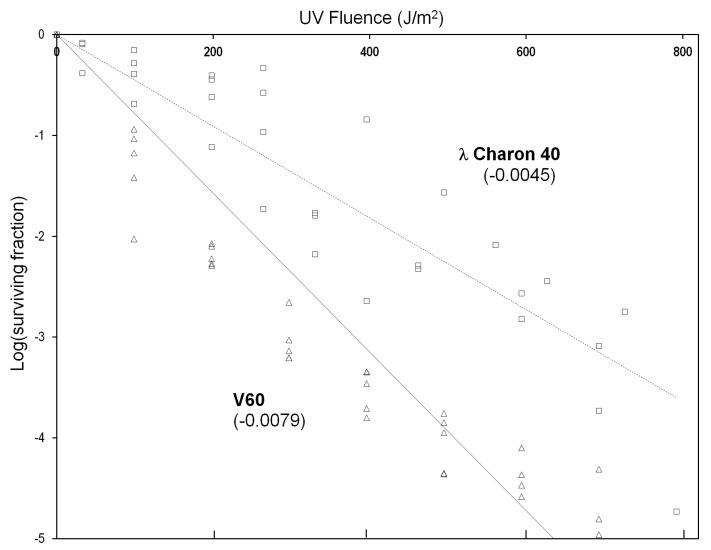
Virion inactivation by UV-C radiation. Virion suspensions in UV-transparent buffer (SM for λ [30], S_dil_ for V60) were irradiated with agitation at room temperature in the dark with a germicidal lamp. The indicated doses represent the UV-C component of the lamp output (wavelengths < 300 nm). Aliquots were withdrawn as indicated and assayed by plaque counting (see Section 2). Numbers in parentheses are first-order rate constants calculated as the slopes of linear regressions of all plotted points, as shown in the graph. Limiting the V60 data to the first four UV doses yields a slope of −0.0097 m^2^/J.

To characterize the ability of the virion structure to stabilize viral DNA, we compared isolate V60 (36 kbp) to the non-lysogenic bacteriophage λ construct Charon 40 (48 kbp) with respect to inactivation by UV-C radiation (see Section 2). As shown in Figure 4, survival of both viruses exhibited approximately exponential decay with respect to UV dose, although V60 exhibited a slightly biphasic survival curve. The apparent first-order dose coefficient for inactivation was at least 1.75 times greater for V60 than for λ, corresponding to approximately 2.4-fold higher UV sensitivity when normalized for genome length.

The slightly higher UV sensitivity of V60 relative to λ indicates that the A conformation of SIRV genomic DNA and its tight binding to the major capsid protein [10] does not reproduce the level of UV protection seen in bacterial endospores, which share these two molecular features with SIRV2 [28]. The result does not preclude the possibility that a modest stabilization of the SIRV DNA is masked or offset by other factors, such as a lower efficiency of repair or bypass of DNA photoproducts in the infected host cell. *S. acidocaldarius*, for example, is killed by UV-C at about twice the rate of *E. coli* [29] even though its genome is less than half as large (2.2 vs. 4.6 Mbp); this suggests correspondingly more effective UV damage, or less effective repair and tolerance, in *Sulfolobus* relative to *E. coli*.

Stability also was assessed by treatments capable of denaturing or otherwise damaging the protein components of the virions. High temperature, for example, disrupts normal protein conformation, and, consistent with adaptation to a geothermal habitat, infectivity of the new isolates was extremely thermostable. Although no PFU survived autoclaving (15 min at 121 °C), 80 °C for one hour caused no apparent loss of infectivity. This contrasted sharply with >99.8% inactivation of bacteriophage T4 after two minutes at 75° and bacteriophage λ after four minutes [31].

**Figure 5:**
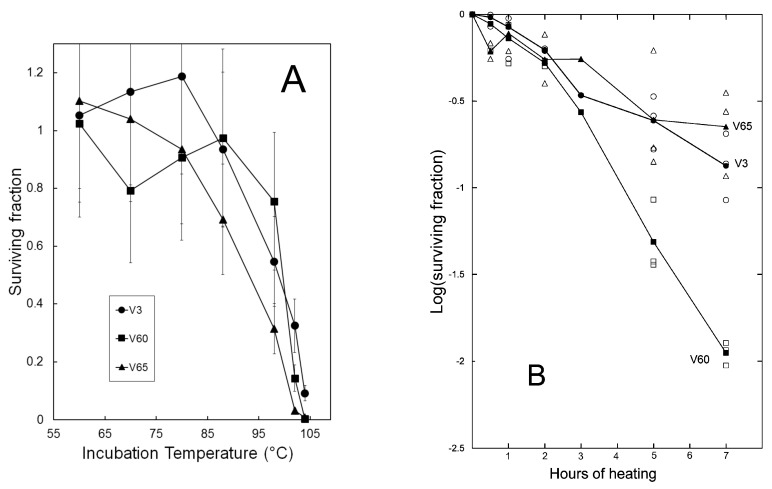
Thermal inactivation of virions. Lysates were diluted to titers of 10^4^–10^5^ per mL in S_dil_ buffer and exposed to the temperature indicated. Surviving PFU were enumerated by plaque assays. Panel (**A**): effects of one-hour incubation at the indicated temperatures (average of three determinations); panel (**B**): time course of infectivity loss in a boiling water bath (solid symbols indicate average of three independent trials).

One-hour exposure to temperatures up to 102 °C allowed thermostability of isolates to be compared (Figure 5A). Based on the results, time-course experiments were used to measure rates of inactivation at a temperature of about 100 °C. Decay rates appeared to vary among the three isolates tested (Figure 5B), but in all cases were much less than those of the most thermostable of 115 *Thermus thermophilus* bacteriophage isolates evaluated by Yu et al. [32]. To confirm that differences in decay rates did not simply reflect variation among individual lysates, independent lysates of each isolate were assayed for survival after 7 h (Table S3). Although survival values varied among individual lysates, chi-square tests confirmed differences among the three viral strains (Table S3), identifying V60 as the least thermostable and V3 as the most thermostable under these conditions.

**Figure 6:**
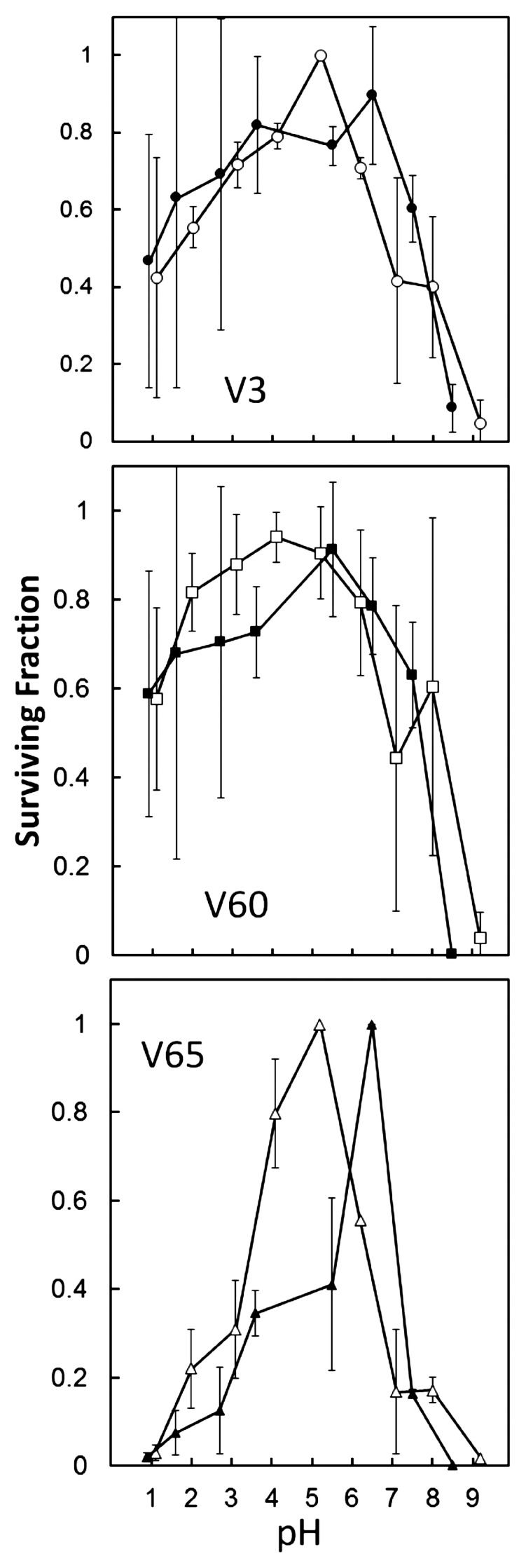
Virion stability as a function of pH. Virus suspensions were diluted in various pH buffers and incubated 70 °C for one hour. For each virus, the two lines (solid vs. open symbols) represent different series of buffers covering similar pH ranges (see Section 2).

To measure stability as a function of pH, concentrated virion suspensions were diluted into various pH buffers and incubated 1 h at 70 °C, followed by cooling, further dilution in *Sulfolobus* dilution buffer (S_dil_), and enumeration of PFU by plaque assay. The results (Figure 6) indicate that maximal virion stability was retained under mildly acidic conditions, i.e., pH 4–6. The chemical composition of the buffer mixture had limited influence on stability, as indicated by the closed vs. open symbols in panels of Figure 6, whereas the overall profiles of pH-dependence of stability tended to distinguish V65 from V3 and V60 (Figure 6). 

The impact of other chemical treatments were evaluated using a similar experimental approach. Chemicals which appeared to yield different survival rates for V3, V60, and V65 included chloroform, urea, guanidinium chloride, and NaIO_4_ (Table 2). Since IO_4_^−^ oxidizes vicinal HCOH groups and cleaves the C-C bond between them [33], this last result suggested that, to the extent the composition of these virions resembled that of SIRV2 [16], carbohydrates attached to one or more of the capsid proteins are important for structural stability or some aspect of the infection process. In initial tests, three treatments (2.75 M guanidinium thiocyanate, 5% SDS, and 5% CTAB) yielded < 1% survival after 1 h, whereas low-specificity proteases (trypsin, Proteinase K, and Pronase) yielded > 25% survival and were not investigated further. Similarly, succinic anhydride, which covalently modifies primary amino groups and reverses their charge, did not cause measurable inactivation, even at 0.1 M and a treatment temperature of 70 °C.

### 3.3. Evaluating Population-Level Variation

The apparent ability of certain treatments to distinguish among the SIRV-like isolates raised questions about the extent to which parameters of virion stability could vary across a population. To increase the size and potential phenotypic diversity of our population sample, we conducted a second culturing survey of the original site and additional sites. In addition to providing new viral isolates for functional characterization, this survey revealed patterns of host and virus distribution among geothermal sites (Table 3). For example, many sites with favorable T and pH values yielded no culturable cells; similarly, detectable concentrations of PFU occurred in a relatively limited subset of sites yielding potential host cells (Table 3).

A larger set of viral strains that included both previous and new isolates was then screened for thermostability. The additional viral isolates exhibited the plaque morphology of strains V3, V60, and V65 and yielded PCR product for primers specific to an internal region of the highly variable gene encoding the minor capsid protein. The initial screening measured survival in a boiling water bath. Isolates that represented either atypically high or atypically low thermostability by this criterion were then evaluated with respect to survival of other treatments. This two-stage screening procedure resulted in a set of six isolates with significant natural variation of virion stability. Survival of the ethanol treatment, for example, ranged from 100% to 0.3%, and survival of the urea treatment ranged from 86% to 0.003%.

It was less clear, however, whether levels of stability to various stress agents co-varied across this sample of isolates. As measured by ranked stability within the set of isolates, boiling and prolonged incubation in 18% ethanol yielded very similar responses (Table S4), which were confirmed to be statistically significant by the Spearman Rank Correlation test (ρ = 0.943, *p* < 0.025). However, results of the other treatments did not correlate significantly; thus, the relative durability of an isolate toward one treatment generally was not a reliable predictor of its relative durability toward another treatment (Table S4).

### 3.4. Acquired Resistance to Viral Infection

Plaques or other areas of confluent lysis on overlay plates produced by V3, V60, or V65 remained clear at 5 d incubation (Figure 1B), demonstrating that nearly all infected cells were killed. However, isolated colonies emerged from these areas of confluent lysis if incubation were continued for longer periods (>7 d, e.g.,). Clones purified from these emergent colonies survived subsequent viral challenge (Figure S4), demonstrating that the rare survivors retained heritable resistance to the viruses used.

In order to characterize the formation of these virus-resistant variants, we performed fluctuation analysis using parallel cultures of the sensitive host strain K00 16-4 (see Section 2). The measured rate of resistant-cell formation, 4.86 × 10^−4^ per cell division (Table 4), was 100 to 1000 times the spontaneous mutation rates for certain chromosomal genes in *Sulfolobus* and *Saccharolobus* strains [18,34,35,36].

To determine whether the observed resistance was caused by CRISPR spacer acquisition, we amplified the CRISPR leader regions of the original sensitive host and several stably resistant derivatives recovered as survivors of infected host populations, in order to detect possible acquisition of spacers following infection. None of the CRISPR leader regions of resistant clones exhibited enlargement (Figure S5); this result indicated that spacer-repeat acquisition was not the dominant mechanism conferring this resistance to virus infection.

To confirm that the observed resistance was not caused by CRISPR-Cas function, we demonstrated that this resistance does not require prior infection by the virus. Host clones from a series of uninfected independent cultures were tested by replica-plating for resistance to V3, V60, or V65 (see Section 2). Screening of 896 such clones identified six that were confirmed by cross-streaking to be stably resistant to at least one of the three isolates, corresponding to a frequency of 6.7 × 10^−4^ resistant clones per cell for the original populations. This frequency, combined with the average size of the independent cultures screened, yielded an estimated mutation rate of 5.8 × 10^−4^ events per cell division, which is similar to the rates calculated from the fluctuation assays that employed direct selection (Table 4).

In view of the high rate at which resistant clones formed, we also tested for the reverse process, i.e., the formation of virus-sensitive cells in initially resistant clones. Of 821 colonies picked from populations of a virus-resistant K00 16-4 clone, 69 were found to exhibit significant killing by at least one of the three isolates (Figure S6), and sensitivity was confirmed in each case by cross-streaking. The frequency of sensitive clones in this experiment, 0.084, combined with the size of the population that was analyzed, yielded an estimated formation rate of 6.3 × 10^−3^ per cell division (see footnote in Table 4). The apparent phenotypic reversion to virus sensitivity was therefore more frequent than the process of forming resistant variants in initially sensitive clones. The 69 sensitive clones recovered in this screen were found to be sensitive to all three of the genetically distinct isolates V3, V60, and V65. In contrast, forward mutation experiments recovered some host variants resistant to only one of the three isolates tested (Table 4).

Given (i) the high rate at which resistance arose in laboratory cultures, (ii) the expected benefit to a host population of generalized resistance to a lytic virus, and (iii) failure of a recent study to recover SIRV-like viruses from at least one thermal region harboring *S. islandicus* [15], we considered whether generalized resistance to these lytic viruses might become fixed in many local populations of the host organism. As a preliminary test, we screened *Sulfolobus*-like strains previously isolated from geographically well-separated regions for sensitivity to virus strain V60, which was cultured from the SRC site.

The potential host strains, isolated in 1999 and 2000, were scored for virus sensitivity by spotting (10^5^ cells in 2 µL) onto plates spread with dilution buffer (control) or V3, V60, or V65 (10^8^ PFU). Clones forming a confluent spot on the control plate and no growth on one or more of the SIRV-spread plates were scored as sensitive. Virus-sensitive isolates were identified in all sets examined, i.e., from the Pisciarelli solfatara, Pozzuoli, Italy (4 of 8), Kamchatka peninsula of Russia (1 of 5), Lassen Volcanic National Park, California (1 of 9) and multiple sites within YNP (14 of 38). Thus, these results extended those of a study that similarly identified host strains sensitive to North American SIRV-like isolates from various geographic locations worldwide [15].

### 3.5. Cellular Basis of High-Frequency Resistance

Acquired resistance to SIRV infection has been reported previously and documented in mechanistic terms. In one example, SIRV2 was used to select resistant mutants of *Saccharolobus solfataricus* strain P2 [38]. The resistant mutants were found to have IS elements inserted into either of two genes encoding putative pilus-assembly proteins, providing evidence that the corresponding pili are necessary for SIRV2 infection of *S. solfataricus* P2 [26,38]. Although IS transposition can be frequent, the rate of native IS transposition into the *pyrE* gene in a set of *S. islandicus* isolates from diverse world regions averaged 6.7 × 10^−7^ per cell division [18], and thus seemed unlikely to account for production of the virus-resistant clones we observed (Table 4). In order to clarify the role of pilus-related genes in the K00 16-4 phenomenon, we analyzed ORFs of this strain corresponding to the genes inactivated in SIRV-resistant strains of *S. solfataricus* P2 by PCR. All spontaneously resistant K00 16-4 strains we examined had no enlargement of the corresponding pilus genes, and sequencing confirmed that the virus-resistant strains also had no other detectable mutations in these genes.

In a more recent study, a clone of host strain M16.4 (alternative designation for K00 16-4 [19]) selected for survival of infection by the fusellovirus SSV9 exhibited concomitant resistance to SIRV-like viruses isolated from within YNP [39]. The resistant clone had a 6068-bp deletion affecting several genes, including two putative pilin genes: M164_0242 (*pilA1)* and M164_0246 (*pilA2)*, suggesting a role for pili composed of PilA subunits, in a situation analogous to the SIRV-resistant *S. solfataricus* P2. Complementation by cloned genes indicated that loss of both *pilA1* and *pilA2* function was necessary to create the SIRV-resistant phenotype [39]. This mode of resistance also seems difficult to reconcile with the high rates of spontaneous resistance that we observed.

Investigation of published *S. islandicus* genomes revealed that *pilA*1 and *pilA*2 genes occur as inverted repeats separated by an interval which varies in length and gene content among these genomes. This configuration permits reciprocal homologous recombination (crossing over) between the two *pilA* copies to reverse the orientation of the central segment, resulting in potentially rapid switching between two configurations. Both orientations of this interval appear among *S. islandicus* genomes, indicating that such recombinational inversions have occurred (Figure S7). However, PCR confirmed that the *pilA1*-*A2* interval exhibited the original orientation in all virus-sensitive and -resistant K00 16-4 clones tested, indicating that inversion of the region between *pilA1 and pilA2* did not cause the spontaneous virus resistance that we observed.

We also noted that in certain genomes, including M16.4, the nucleotide sequences of the *pilA1* and *pilA2* genes differ, resulting in differences between the encoded proteins. In the case of M16.4, the two genes differ at 26 positions, creating 16 amino acid differences between the encoded pilins (Figure S7). We therefore considered whether crossover events may re-assort (i.e., swap), sections of *pilA1* and *pilA2* to create a recombinant allele not supporting virus infection. In principle, the hypothesized recombination events could be both frequent and reversible, thereby accounting for the observations that seemed inconsistent with previously described SIRV-resistance mechanisms. Sequence analysis of *pilA1 and pilA2* showed, however, that neither gene was altered in any of the resistant K00 16-4 variants, effectively eliminating mutation of a *pilA* gene as the primary mechanism of the high-frequency resistance we observed in this strain.

To characterize the cellular basis of the acquired resistance more directly, we tested the ability of sensitive and resistant strains to bind infective virions. As described in Section 2, virions were added to a numerical excess of cells and incubated at a physiological temperature (70 °C). At multiple time points, each mixture was diluted in cold buffer to retard further adsorption, and was centrifuged to separate cell-bound forms of the virus from free (unadsorbed) virions. These two forms were then quantified separately by sampling and plaque-assay of supernatant and washed-pellet fractions. The unfractionated mixtures (and fractions of a control tube without cells) provided estimates of the total (and background) PFU at each time point (see Section 2).

**Figure 7:**
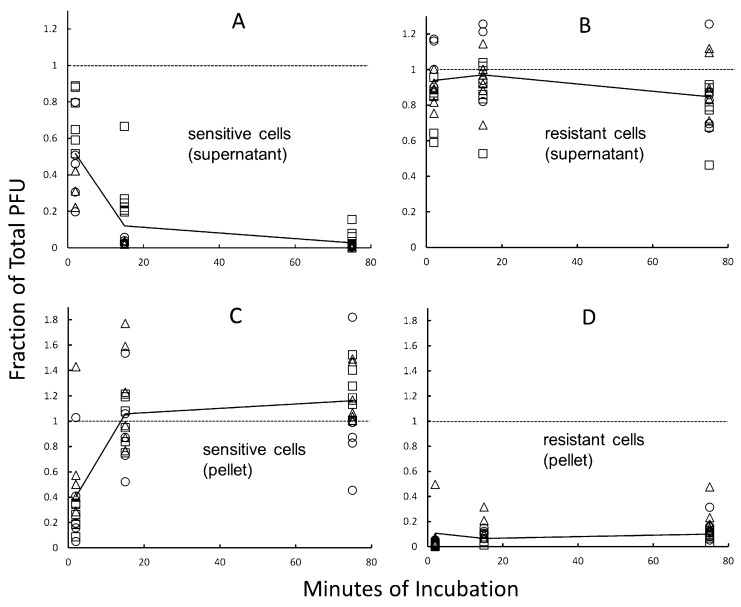
Virion binding. V3 (circles), V60 (squares) or V65 (triangles) were added at low MOI to either of two resistant clones of *S. islandicus* K00 16-4 (panel (**A**,**C**)) or two sensitive revertants of spontaneously resistant clones (panel (**B**,**D**)), and the resulting mixtures were as described in Section 2. Each data point depicts an independent, individual plaque count normalized to the number of PFU added to the cell suspensions (indicated by the dotted horizontal line); samples of unfractionated cell/virus mixtures yielded an average of 420 plaques under these conditions. Independent assays were performed in triplicate for each combination of host and virus strain, and the average values for all combinations are connected by a solid line. Pellet fractions are corrected for the average number of PFU in parallel sham (no-cell) controls, which was 1 plaque.

As summarized in Figure 7, virus-sensitive cells depleted free virions from the mixed suspensions in a time-dependent manner, resulting in nearly quantitative removal of PFU from the supernatant fractions and corresponding recovery in the pellet fractions. Virus-resistant cells, in contrast, were inefficient at binding virions. Isolates V3, V60, and V65, represented by distinct symbols in Figure 7, gave similar results for both types of host cell (sensitive vs. resistant). In some individual assays, PFU were recovered from the pellet fractions of resistant strains, increasing with time of incubation. The yield of these cell-associated PFU varied markedly among replicates, however and was always much lower than the corresponding value for virus-sensitive strains (Figure 7D vs. Figure 7C). One potential explanation for this result is spontaneous reversion during the growth of resistant cultures (see above). Alternatively, if partial impairment, rather than complete inactivation, of virion binding would suffice to create a virus-resistant phenotype in host-cell populations, similar experimental results may have been observed.

## 4. Discussion

Although lytic archaeal viruses represent relatively few described species of the diverse viruses of hyperthermophilic archaea, examples have been isolated from multiple geothermal regions worldwide [8,13,15]. Our results and those of others further indicate that lytic viruses resembling SIRVs form localized populations that can remain associated with specific hydrothermal features for periods of several years. This is illustrated by the SRC site, where a hydrothermal feature maintained high concentrations of SIRV-like viruses over a six-year period, and an area within the Norris Geyser Basin, from which two groups independently isolated multiple SIRV-like strains over a four-year period [15]. At both sites, viral populations (as measured by PFU) were smaller and more spatially focused than those of potential hosts (as measured by CFU) (Table 4). Analysis of genomes (Table S2) indicated that the new isolates are most closely related to other SIRV-like viruses of *S. islandicus* isolated from YNP [15]. Geographically patterned similarity was evident also in the conservation of protein-encoding genes, as was observed previously for the isolates from other regions within YNP [15]. For example, the new isolates lacked several genes shared by SIRV1 and SIRV2 (Table S2). The genetic diversity recovered by limited sampling of three sub-populations within YNP [15], combined with the broad distribution of virus-sensitive hosts in geothermal regions throughout the world, illustrates the potential size and complexity of a global network of meta-populations of SIRV-like lytic viruses.

While diversity of archaeal viruses has been well documented in terms of genome sequences, relatively few functional properties of these viruses have been analyzed. The present study is, to our knowledge, the first to examine virion stability and its natural variation, and the results provide new data regarding the environmental challenges to the propagation of these lytic viruses. Isolate V60 was found to be slightly more UV-sensitive than bacteriophage λ, despite its smaller genome size. This has potential implications for properties of DNA observed in SIRV2 and expected to be shared by other rudiviruses, specifically, (i) tight association with a structural protein, and (ii) the A conformation of the DNA helix. Both of these unusual characteristics are shared by SIRV2 and bacterial endospores [10], yet our results suggest that in SIRVs they do not cause the dramatic attenuation of photoproduct formation seen in endospores [28].

With respect to temperature, the SIRV-like isolates were extremely stable compared to bacteriophages, including the *Thermus* bacteriophage [32], while some differences in temperature- and pH-stability parameters could be observed among isolates. Certain chemical treatments did not distinguish among the viral isolates, although the impacts differed dramatically among treatments. An amino-modifying agent had little effect, for example, whereas periodate inactivated the virions. The results of these various treatments seem consistent with what is known about the molecular architecture (including protein glycosylation) of SIRVs and cell surfaces of Sulfolobales [16,40,41]. The limited impact of non-specific proteases may result, in part, from the protective effects of glycosylation, as observed for S-layer proteins of *Sulfolobus*, for example, whereas inactivation by periodate under otherwise mild conditions provides experimental evidence that the glycosylation state of virion protein(s) is important for structural stability, some aspect of the infection process, or both [40,41]. Similarly, insensitivity to inactivation by succinic anhydride is consistent with protein-modification studies of SIRV2 [16] and the steric and electrostatic shielding of the amino terminus of the major capsid protein evident in high-resolution 3-D reconstructions [10].

Other chemical treatments (i.e., chaotropes and organic solvents) revealed additional examples of natural variation of virion stability. Nevertheless, even the evaluation of a larger, more diverse set of isolates detected few correlations of stability properties, which was unexpected, given the large differences in stability toward many of the treatments. This limited co-variation suggests that stability determinants may vary relatively independently, which may reflect the complexity of molecular interactions required for maximal stability of these virions. The situation seems particularly striking in view of the fact that the 134-amino-acid major capsid protein, which mediates both DNA binding and helix formation in the mature virion, has the same amino acid sequence in V3, V60, V65 (Figure S3) and the other SIRV-like isolates from YNP that have been analyzed by sequencing [15]. Thus, the stability differences among V3, V60, V65 (and perhaps the additional lytic isolates we tested) cannot be attributed to the primary structure of the major capsid protein responsible for the basic virion structure. On one hand, this situation highlights our limited understanding of specific molecular features that preserve the biological function of viruses at high temperature and low pH. On the other hand, it suggests the possibility of experimentally identifying these yet-unknown molecular features that contribute to the impressive stability of these simple virions.

Our results also revealed new insight into phenotypic variation of hosts and the potential complexity of molecular mechanisms that limit the killing of archaeal cells by lytic viruses. Resistant clones of a commonly used *S. islandicus* strain formed at much higher rates than predicted by spontaneous gene mutation, whereas reversion back to a virus-sensitive phenotype occurred at even higher rates, and none of the genetic mechanisms known to confer resistance to SIRVs could be implicated in these two reciprocal conversions. The virus-resistant strains selected by infection, for example, did not acquire new CRISPR spacer-repeats, and formed at the same rate without any prior exposure to the virus. Similarly, two distinct clusters of pilus-related genes responsible for spontaneous resistance to SIRV2 [38] or a related isolate from YNP [39] were found to be intact in all the resistant strains we analyzed. Thus, our results indicate that a yet-unidentified mechanism triggers relatively rapid inter-conversion of virus-sensitive and -resistant hosts.

Although its genetic basis remains to be identified, the mode of resistance we observed nevertheless could be attributed experimentally to impaired physical attachment of virions to host cells. In this respect, the mode of resistance therefore may resemble previously reported examples of SIRV-resistance that involve loss of certain pili [38,39]. Gain or loss of resistance to one virus strain generally applied to other strains, with the exception of some non-selected resistant variants, which remained sensitive to some of the viral isolates tested. The basis of this apparent specificity remains an open question requiring further study.

Regardless of the mechanism and specificity of inter-conversion between sensitive and resistant forms, its high rate seems likely to play an important functional role in stabilizing the predator-prey relationship between SIRV-like lytic viruses and their hosts in nature. The predicted effect of the observed spontaneous inter-conversion would be to maintain a proportion of a host population in the sensitive state and the remainder in the resistant state regardless of the population’s previous history. This mode of resistance to infection thus should create a form of ‘bet-hedging’ that would act to ensure survival of both virus and host whenever a local host population is infected. This process (or a functionally equivalent one) may account for the stable carrier states that have been reported for SIRV3-infected host strains under laboratory conditions [42].

Analogous switches, which include bi-stable regulatory networks and highly mutable genes termed contingency loci, have been found to play ecologically important roles in various bacteria and to be stabilized by selection [43,44]. Although most of the described systems are host-encoded, the genetic switches controlling the lysis vs. lysogeny decision of bacteriophage λ and the host range of bacteriophage Mu [45,46] represent classic virus-encoded examples of such mechanisms, and have parallels in archaeal viruses [2]. Retention of such genetic switches by diverse viruses provides evidence that corresponding forms of ‘bet-hedging’ can benefit viruses’ ability to persist in natural host populations.

## Figures and Tables

**Table 1:** Summary of Virus Isolations.

Designation	Origin	Reference
SIRV1	Iceland, Kverkjoll solfatara	[12]
SIRV2	Iceland, Hveragerdi solfatara	[13]
SIRV3	Iceland, Gunnuhver solfatara	[14]
SIRV4–7, 11	Wyoming (USA), Nymph Lake	[15]
	(44.7519, −110.7286) ^a^	
SIRV8–10	Wyoming (USA), Norris Geyser Basin	[15]
	(44.7278, −110.7139) ^a^	
V1–65	Wyoming (USA), Midway Geyser Basin	this work
	(44.5089, −110.8089) ^a^	
Y11 40.32	Wyoming (USA), Midway Geyser Basin	this work
	(44.5089, −110.8089) ^a^	
Y11 27.5	Wyoming (USA), Norris Geyser Basin	this work
	(44.7286, −110.7134) ^a^	

^a^ Latitude and longitude co-ordinates marking the approximate site of the cluster of features sampled within the named geothermal region.

**Table 2:** Virion inactivation by chemicals.

	Surviving Fraction (Std. Dev.)		
Treatment	V3	V60	V65
NaIO_4_	0.0247 (0.0189)	0.0016 (0.00074)	0.00014 (3.8 × 10^−5^)
CHCl_3_	0.0210 (0.0189)	0.0338 (0.0126)	0.180 (0.248)
Urea	0.0067 (0.0075)	0.0071 (0.0092)	0.0503 (0.0690)
Guanidine HCl	0.219 (0.222)	0.544 (0.564)	0.0010 (0.0012)

Virion suspensions were treated for one hour with 0.25 M NaIO_4_ or chloroform at 37 °C, or 5 M urea or 4.5 M guanidinium chloride at 70 °C, followed by quenching, dilution, and plaque assay (see Section 2). Values are the average ratio of the resulting titer to that of a corresponding control from three independent assays.

**Table 3:** Results of environmental culturing survey.

Thermal Area	Sample No.	pH	Temp., °C	CFU/mL	PFU/mL
Ebro Springs					
	1	3.5	58	<10	<5
	2	-	57	<10	<5
	3	-	58	<10	<5
Washburn area					
	4	2.4	71	<10	<5
	5	4.2	69	<10	<5
	6	3.2	69	550	<5
	7	3	76	160	<5
	8	3.3	87	170	<5
	9	3.1	76	200	<5
	10	2.5	78	<10	<5
	11	2.6	82	20	<5
	12	2.8	73	110	<5
	13	3	61	30	<5
Norris Geyser Basin					
	14	5	83	270	<5
	15	3.5	86	<10	<5
	16	3.9	69	5000	<5
	17	3.2	76	12,000	<5
	18	3.2	83	140	<5
	19	3	77	500	<5
	20	3	83	25	<5
	21	2.7	89	<10	<5
	22	3	59	5000	<5
	23	3	82	<10	<5
	24	2.7	82	<10	<5
	25	3.1	77	7000	<5
	26	3	82	<10	<5
	27	2.5	70	10,000	155
	28	3	75	2200	25
	29	3	60	<10	<5
	30	3	70	900	<5
	31	4.5	79	3800	<5
	32	2.7	61	<10	<5
	33	2.7	73	<10	<5
	34	2.4	84	<10	<5
	35	2.4	84	<10	<5
	36	4.7	81	<10	<5
	37	4	72	<10	<5
	38	4	80	5500	<5
Rabbit Creek area					
	39	4	81.5	30,000	50
	40	2.4	80	40,000	170
	41	3.9	82	30,000	<5
	42	3.9	82	35,000	<5
	43	-	81	14,000	5
	44	3.9	78	47,000	<5
	45	-	81	30,000	5
	46	4	79	20,000	<5
	47	-	74	30,000	<5
	48	3.9	69	47,000	<5
	49	-	66	97,000	<5
	50	3.5	80	5000	<5
	51	-	69	650	<5

Results for direct plating of samples collected in 2011. Latitude and longitude co-ordinates of locations: Ebro Springs: 44.5879, −110.3410; Washburn area: 44.7649, −110.4476; Norris Geyser Basin: 44.7280, −110.7143; Rabbit Creek: 44.5089, −110.8089.

**Table 4:** Rates of Resistant Cell Formation.

**Selected Resistance ^a^**		
Challenge Virus	**µ**	95% Confidence Limits
V3	4.47 × 10^−4^	2.87, 8.49 × 10^−4^
V60	3.46 × 10^−4^	2.48, 5.28 × 10^−4^
V65	6.65 × 10^−4^	4.17, 8.02 × 10^−4^
Mean	4.86 × 10^−4^	
**Non-Selected Resistance ^b^**		
Resistant to	Number Observed	
V3 only	1	
V60 only	3	
V65 only	0	
All three	2	
(Total: 6)		

^a^ Results of 20 independent cultures (see Section 2) subjected to maximum-likelihood analysis [24]. ^b^ Results of replica-plating 896 clones drawn randomly from a population of *n* = 1.4 × 10^8^ cells, yielding a corresponding frequency (f) of resistant variants in the population of 6.7 × 10^−4^. Solving the equation µ = f/ln(Nµ) by iteration [37] yields µ = 5.84 × 10^−4^ conversions per cell division.

## Data Availability

Genome sequences are deposited in GenBank under the following accession numbers: isolateV3, OM401712; isolateV60, OM401713; isolateV65, OM401714.

## Supplementary Materials

The following supporting information can be downloaded at: https://www.mdpi.com/article/10.3390/v14071439/s1, Table S1. PCR primers; Table S2. Genes conserved among Icelandic and North American isolates; Table S3. Statistical comparison of virion thermostability; Table S4. Stability properties of an expanded population sample; Figure S1. Test for renaturation of viral restriction fragments; Figure S2. Comparison of viral genomes; Figure S3. Major capsid protein sequences; Figure S4. Scoring resistance by cross-streaking; Figure S5. Testing for enlargement of CRISPR loci; Figure S6. Acquired sensitivity to virus infection; Figure S7. Genetic organization of *S. islandicus pilA1-pilA2* region
