# Supplementary material for: Diversity of SIRV-like Viruses from a North American Population
Source: Viruses. 2022 Jun 30;14(7):1439. doi: 10.3390/v14071439 (PMC9319562; doi:10.3390/v14071439)

Supplementary Material for Fackler *et al., 2022* (Tables S1–S4, Figures S1-S7)

Table S1. PCR primers

Region Analyzed	Primer Name	Primer Sequence
M16.4 (K00 16-4) CRISPR 1 leader region		
	16.4 C1 MidFor 1	AAAAATTACACGGTATCTGGGATC
	16.4 C1 MidFor 2	CTCATTTCTAGGGTTGAAATAAGGTAC
	16.4 C1 Rev	TAAGGAGTGGGGTAAAGACATTC
M16.4 (K00 16-4) CRISPR 2 leader region		
	16.4 C2 For	CATTAAATCCCATGGTCACAAAATATG
	16.4 C2 MidRev 1	ATAATCTAAAGGGAGAAGAAAGAATCAAG
	16.4 C2 MidRev 2	ATTCCATTATTTCTCAGAATATCAGAAAAG
M16.4 (K00 16-4) Homologues of *S. solfataricus* P2 pilus genes		
	M164_0199 FWD1	CTCACATGTTCTAGAAGATAAAGA
	M164_0199 REV1	GACCTATTGTAAATGCTAATACG
	M164_0199 FWD2	GCATACAAGATATTTTACGTTAC
	M164_0199 REV2	CTGGTGGTTCTAATAAAACAT
	M164_0200 FWD1	GCAGACTATGAAGTGAATATACTAA
	M164_0200 REV1	TTCATCATAGGTATCCACTCTT
	M164_0200 FWD2	ATAGTAGTAGGCGAGATAAGA
	M164_0200 REV2	GTTTAAATGGAAGGTAACTCC
	M164_2227 FWD1	AACAGTAACGATTATTATCAACAAA
	M164_2227 REV1	TGCACCTAAAACAACTTGAA
	M164_2228 FWD1	CTTCTCAAGCTAATTTCTATTTTC
	M164_2228 REV1	ACATTATACCGTAATTCCCTC
	M164_2228 FWD2	GTGGTAACTTCGAAATTGTT
	M164_2228 REV2	TTGGGACTGTTGTGTAGTAT
	M164_2228 FWD3	ATGGGCAATAGCAATATCAATA
	M164_2228 REV3	GGAATAGTCAAGATAAGGTCAT
M16.4 (K00 16-4) putative pilin genes		
	16-4 PilA1 fwd	AATAAGTTGAGGTGCCATT
	16-4 PilA1 rev	ATTTGTTGGATGAGGGTAG
	16-4 PilA2 fwd	ATTCTGACTTATCGGGAAT
	16-4 PilA2 rev	CTCACTAGCGTATTGTTGC

Table S2. Genes conserved among Icelandic and North American isolates[a]

SIRV1		SIRV2		V3		V60		V65			
ORF	AA	ORF	AA	Location	AA	Location	AA	Location	AA	dN/dS	Functional Feature[b]
gp19	134	gp26	134	9903-10307	134	8495-8899	134	8199-8603	134	0.083	major capsid protein
gp21	335	gp28	335	10554-11561	335	9146-10153	335	8850-9857	335	0.209	group 1 glycosyl transferase
gp36	356	gp44	356	26564-27628	354	26116-27180	354	24818-25804	328	0.360	group 1 glycosyl transferase
gp27	121	gp35	121	16641-17006	121	15233-15622	129	14937-15302	121	0.401	Holliday junction resolvase
gp06	306	gp13	310	29256-30191	311	28808-29743	311	27024-27959	311	0.421	tRNA ribosyl transferase
gp10	131	gp17	131	7096-7497	133	5484-5885	133	5404-5805	133	0.501	ssDNA-binding protein
gp42	98	gp49	98	8280-8567	95	6676-6963	95	6596-6895	99	0.681	virion release structure
gp07	399	gp14	399	28063-29253	396	27615-28805	396	25831-27021	396	0.520	amino-acid transporter
gp31	562	gp39	562	20762-22462	566	20434-22134	566	19058-20758	566	0.852	structural protein
gp25	510	gp33	462	14299-15699	466	12891-14291	466	12595-13995	466	0.877	structural protein
gp11	440	gp18	436	5763-7070	435	4151-5458	435	4071-5378	435	0.918	ssDNA annealing
gp35	209	gp43	176	25843-26448	201	25395-26000	201	24019-24624	201	1.07	glycosyl transferase
gp33	158	gp41	158	24123-24605	160	23675-24157	160	22299-22781	160	1.11	methyl transferase
gp30	1070	gp38	1070	17497-20673	1058	16329-19505	1058	15793-18969	1058	1.22	minor capsid protein
gp12	207	gp19	207	5142-5760	205	3531-4148	205	3451-4068	205	1.49	ssDNA nuclease
gp09	125	gp16	119	7909-8286	125	6297-6674	125	6217-6594	125	1.79	replication initiator protein

[a] Homologus open reading frames shared by the five viruses are listed in order of increasing dN/dS ratio. The following ORFs of SIRV1 and -2 were not found in the genomes of YNP isolates: SIRV1 gp05, gp12, gp15, gp29 and gp44, corresponding to SIRV2 gp12, gp20, gp22, gp37, and gp52, respectively.

[b] Based on analyses of Bautista MA, Black JA, Youngblut ND and Whitaker RJ (2017) Viruses 9:10.3390/v9050120; Oke M, Kerou M, Liu H, Peng X, Garrett RA, Prangishvili D, Naismith JH, White MF. 2011. J. Virol. 85:925-931. doi: 10.1128/JVI.01467-10; Guo, Y, Kragelund BB, White MF, Peng X. 2015. J. Mol. Biol. 427:2179-2191. doi: 10.1016/j.jmb.2015.03.013 [doi]; Prangishvili, D, Koonin EV, Krupovic M. 2013. Biochem. Soc. Trans. 41:443-450. doi: 10.1042/BST20120313 [doi].

Table S3. Statistical comparison of virion thermostability[a]

	V3	V60	V65
	-1.146	-2.359	-2.642
	-1.126	-2.271	-1.690
	-1.101	-2.084	-1.664
	-1.046	-2.013	-1.623
	-1.000	-1.889	-1.615
	-1.000	-1.824	-1.560
	-0.884	-1.820	-1.523
	-0.824	-1.760	-1.512
	-0.821	-1.745	-1.386
	-0.799	-1.723	-1.382
	-0.747	-1.699	-1.262
	-0.740	-1.664	-1.212
	-0.708	-1.601	-1.117
	-0.553	-1.557	-0.915
	-0.420	-1.529	-0.873
		-1.507	
**n[b]**	15	16	15
**Mean**	-0.861	-1.815	-1.465
**Median**	-0.824	-1.752	-1.512

[a]Values in each column are log(final titer/initial titer) for 7-h incubation in a boiling water bath, listed in ranked order.
[b]Independent replicate measurements of six lysates per virus; two-tailed T tests yield P values of 3×10^{-12}, 0.01, and 6×10^{-5} for V3 *vs*. V60, V60 *vs*. V65, and V65 *vs*. V3, respectively.

Table S4. Stability properties of an expanded population sample.

Survival values are log(surviving fraction) averaged over three assays. Treatments: 2 h in boiling water bath, other treatments were at 60° C (1 h in 5 M urea, 1h in 5 M guanidinium chloride, 30 min in chloroform-saturated buffer, 36 h in 18% ethanol); "SD" indicates standard deviation.

**Isolate**	**Boiling**			**Urea**			**Guanidinium**			**$CHCl_3$**			**Ethanol**			**Overall Rank**	
	Survival	SD	Rank	Survival	SD	Rank	Survival	SD	Rank	Survival	SD	Rank	Survival	SD	Rank	avg	SD
V36	-0.060	0.013	1	-0.593	0.016	5	-1.259	0.102	3	-1.540	0.088	6	0.011	0.030	1	3.2	2.04
V55	-0.101	0.031	2	-0.534	0.266	4	-0.383	0.089	2	-0.912	0.301	2	-0.022	0.002	2	2.4	0.8
40.32	-0.218	0.217	3	-0.064	0.063	1	0.017	0.100	1	-1.228	0.058	4	-0.033	0.050	3	2.4	1.2
27.5	-0.865	0.931	4	-0.282	0.103	3	-1.262	0.068	4	-1.283	0.091	5	-1.287	0.098	5	4.2	0.75
27.16	-1.879	0.235	5	-0.174	0.128	2	-1.369	0.091	6	-0.413	0.345	1	-0.039	0.020	4	3.6	1.85
V45	-1.901	0.603	6	-4.460	0.337	6	-1.361	0.104	5	-0.932	0.556	3	-2.534	0.549	6	5.2	1.17

Figure S1. Test for renaturation of viral restriction fragments.

Purified V3 or λ DNA (100 ng) was digested with endonuclease StyI 1h in NEB1 buffer at 37° C. Half of the resulting digest was heated to 100° C for 5 min and then chilled quickly to 0° C, and the resulting two samples were electrophoresed in 1% agarose in adjacent lanes. White arrowheads and numbers beside the gel photograph indicate length of individual restriction fragments. The 10.1 and 6.2 kbp StyI fragments of V3 DNA are the only StyI fragments that renature under these conditions, and correspond to the two ends of the linear V3 genome sequence. Lane A, V3 unheated; lane B, V3 heated and cooled; lane C, λ unheated; lane D, λ heated and cooled.

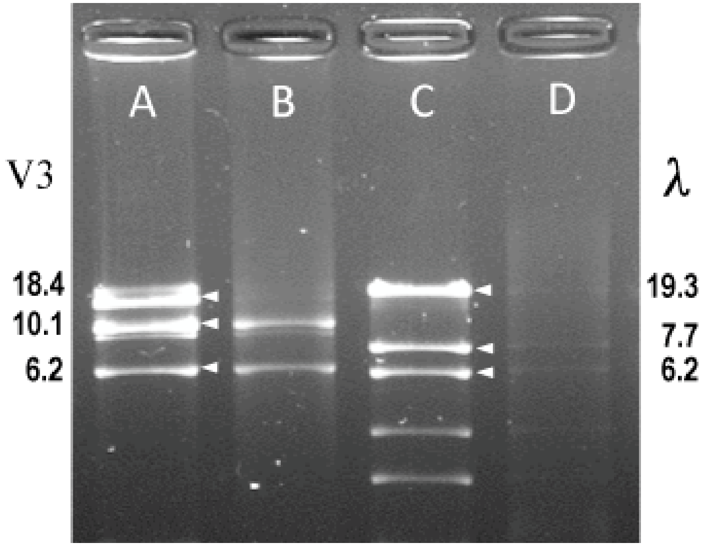

Figure S2. Comparison of viral genomes

Genome sequences of the North American (V3, V60, V65) and Icelandic (SIRV1, SIRV2) isolates were aligned by Progressive Mauve (see Methods). Blocks of corresponding sequences shared among the isolates are indicated.

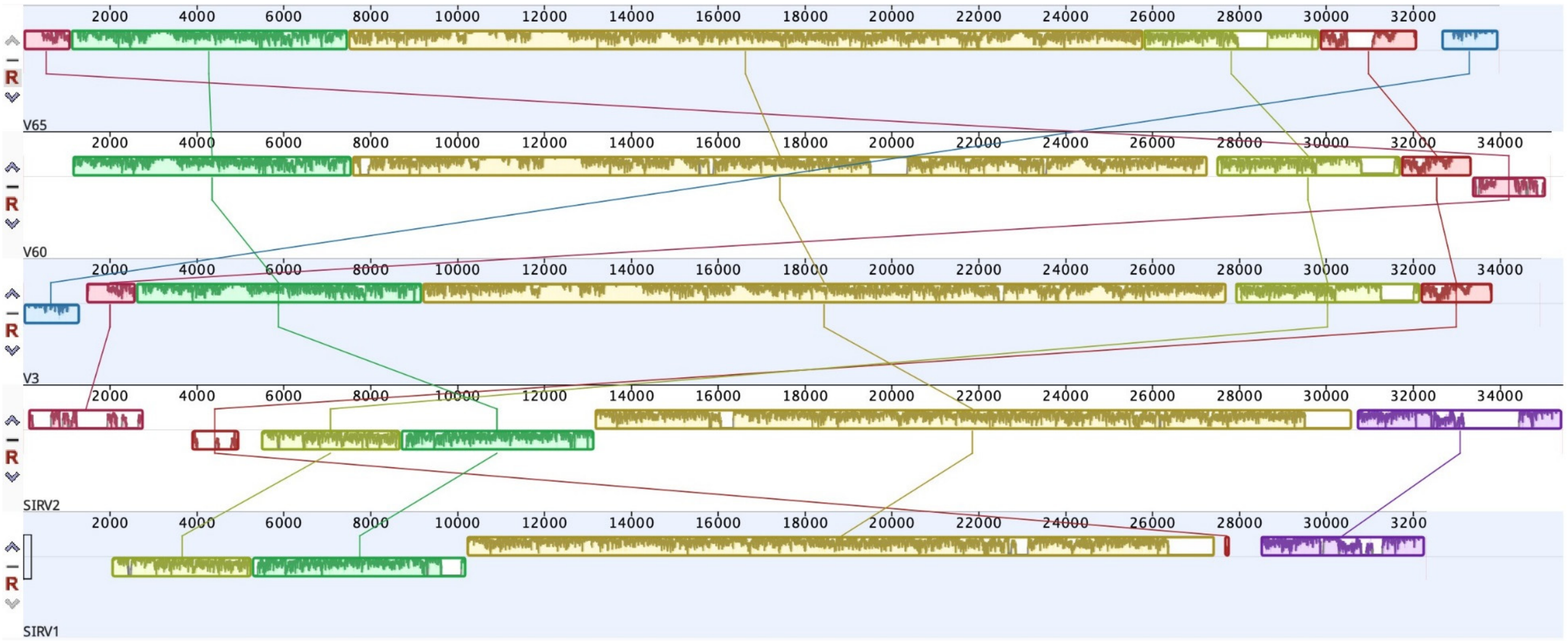

Figure S3. Major capsid protein sequences.

Sequence of the gene encoding the major capsid protein; the consensus sequence indicates 90% (uppercase) and 50% (lowercase) agreement. V3, V60, and V65 differ from each other at 35 nucleotide positions (marked by asterisks) but encode the same polypeptide (MAKGRTPRSYSQRYAKWNAKFVSFSNPTVASTILSNVAPVAQQNFQTNVPKFTSVNEQVSAVLSEYGITGPNRAIYQGFGLKVARALNRLGGGPALVNMINGLKAYYISAFNANPTVLDAVTNIITGSPTGYVS).

```
           1                                                                                                         100
       V3  ATGGCAAAAG GAAGAACACC AAGATCATAT AGTCAAAGAT ATGCTAAGTG GAATGCAAAA TTTGTATCAT TTTCAAATCC AACTGTCGCT AGCACTATAT
      V60  ATGGCAAAAG GAAGAACACC AAGATCATAT AGTCAAAGAT ATGCAAAATG GAACGCTAAG TTTGTCTCAT TTAGCAATCC AACTGTAGCT AGTACAATAT
      V65  ATGGCAAAAG GAAGAACACC AAGATCATAT AGTCAAAGAT ATGCAAAATG GAACGCTAAG TTTGTCTCAT TTAGCAATCC AACTGTAGCT AGTACAATAT
    SIRV1  ATGGCAAAAG GTCACACAAA AAGATCATAT AGCCAAAGGT ACGCAAAATG GCAAGCCAAA TTTAATGCGT TTAGTAACCC AACAGTAGCA TCTACTATAT
    SIRV2  ATGGCAAAAG GTCACACATC AAGAAGTTAT TCACAAAGAT ATGCAAAATG GCAAGCTAAG TTTAATGCAT TTTCAAATCC AACTGTCGCT AGCACTATAT
Consensus  ATGGCAAAAG GaagaACAcc AAGAtcaTAT agtCAAAGaT AtGCaAAaTG GaA.GCtAAg TTTgt.tCaT TTag.AAtCC AACtGTaGCt agtACtATAT
                                                           *  *      *  *  *      *       ***            *      *  *

           101                                                                                                       200
       V3  TGTCAAATGT TGCACCAGTT GCACAACAAA ATTTCCAAAC TAATGTTCCC AAATTCACAT CTGTAAATGA ACAAGTTAGT GCAGTATTAA GTGAGTATGG
      V60  TGTCAAATGT TGCACCAGTT GCACAACAAA ATTTCCAAAC TAATGTTCCT AAGTTCACAT CTGTAAATGA ACAAGTTAGT GCAGTATTAA GTGAGTATGG
      V65  TGTCAAATGT TGCACCAGTT GCACAACAAA ATTTCCAAAC TAATGTTCCC AAATTCACAT CTGTAAATGA ACAAGTTAGT GCAGTATTAA GTGAGTATGG
    SIRV1  TGTCAAATGT AGCTCCTGTT GCCCAACAGA ATTTCCAGAC TAATGTCCCA ACATTTACCG CGGTTAACGA AAATGTTAGT GCAGTTCTTT CACAATATGG
    SIRV2  TATCTAATGT GTCTCCAGTT GCCCAACAAA ATTTCCAGAC TAATGTTCCT AAATTTACCA GTGTTAATGA AAATGTTAGT GCTGTGTTGA CACAATATGG
Consensus  TgTCaAATGT tgCaCCaGTT GCaCAACAaA ATTTCCAaAC TAATGTtCC. AaaTTcACat ctGTaAAtGA AcAaGTTAGT GCaGTatTaa gtgAgTATGG
                                                                *   *

           201                                                                                                       300
       V3  AATAACGGGG CCTAACAGAG CGATTTACCA AGGTTTCGGC CTTAAGGTAG CTAGAGCGCT TAATAGATTA GGCGGTGGAC CAGCACTTGT AAATATGATT
      V60  AATAACGGGG CCTAACAGAG CGATTTACCA AGGTTTCGGC CTTAAGGTAG CTAGAGCTTT AAATAGACTT GGCGGTGGCC CGGCACTAGT TAATATGATA
      V65  AATAACGGGG CCTAACAGAG CGATTTACCA AGGTTTCGGC CTTAAGGTAG CTAGAGCGCT TAATAGATTA GGCGGTGGGC CAGCACTTGT TAATATGATA
    SIRV1  AATAACTGGG CCTAACAGAG CTATATATCA AGGTTTTGGT TTGAAGATTG CTAGAGCACT TAATAGGATT GGGAGCGGGC CAGCTTTAGT AAATATGATA
    SIRV2  AATTACTGGC CCTAATAGAG CAATTTATCA AGGTTTTGGT TTGAAGGTCG CTAGAGCACT TAATAGAATT GGGAGCGGGC CAGCTTTAGT AAACATGATT
Consensus  AATaACgGGg CCTAAcAGAG CgATtTAcCA AGGTTTcGGc cTtAAGgTaG CTAGAGC.cT tAATAGa.Tt GGcgGtGGgC CaGCacTaGT aAAtATGATa
                                                                         **  *      * *         *   *     *   *        *

           301                                                                                                             405
       V3  AACGGTTTGA AAGCATATTA TATTTCAGCG TTTAATGCAA ATCCTACCGT ATTAGATGCA GTTACAAATA TAATTACTGG AAGTCCCACT GGATATGTAA GCTAA
      V60  AACGGTTTGA AAGCATATTA TATTTCAGCG TTTAATGCAA ATCCTACCGT ATTAGATGCA GTTACAAATA TAATTACTGG AAGTCCCACT GGTTATGTAA GCTAA
      V65  AACGGTTTGA AAGCATATTA CATTTCAGCA TTTAATGCAA ATCCTACAGT ATTGGATGCG GTAACTAATA TAATTACTGG ATCTCCTACT GGATATGTAA GCTAA
    SIRV1  AACGGTTTGA AAAGTTATTA TATTTCAGCT TTTAACGCTA ATCCTCAAGT ATTAGATGCT GTAGTAAATA TAATAACTGG AAGCCCGACT GGATACGTAA GTTAA
    SIRV2  AATGGTTTGA AAGGATATTA TATCTCAGCT TTTAACGCAA ATCCACAAGT ATTAGATGCA GTAGTTAATA TCATAACTGG AAGCCCAACT GGATATGTAA GTTAA
Consensus  AAcGGTTTGA AAgcaTATTA tATtTCAGC. TTTAAtGCaA ATCCtacaGT ATTaGATGCa GTaacaAATA TaATtACTGG AagtCC.ACT GGaTAtGTAA GcTAA
                                 *        *                   *      *     *   *  *                  **    *      *
```

Figure S4. Scoring resistance by cross-streaking.
The cross-streak plate was prepared by spreading a virus suspension as a vertical stripe about 2 cm wide in the center of a DT plate. Control and test strains were then smeared from one edge of the plate across the central virus zone. In the results shown, the top four streaks were selected as resistant host variants, and the bottom streak was wild-type K00 16-4, exhibiting the sensitive phenotype.

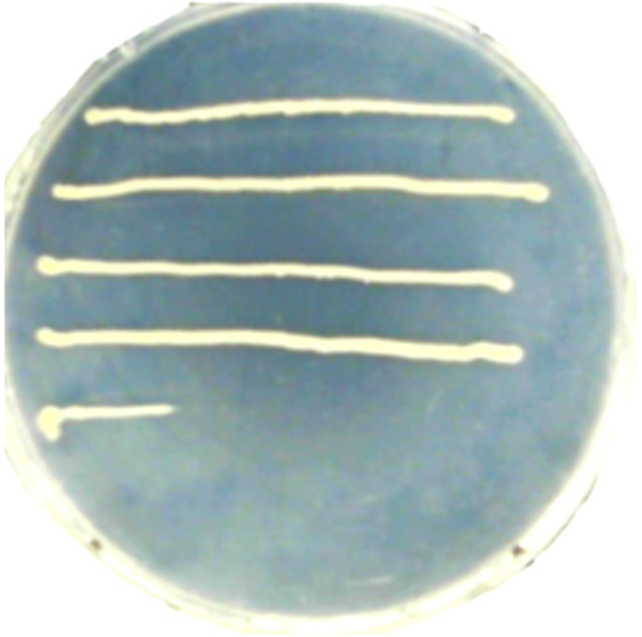

Figure S5. Testing for enlargement of CRISPR loci.

Leader regions of the two CRISPR loci of K00 16-4 were amplified from WT and from purified clones of host selected for virus resistance; primers are listed in Table S1. PCR products from virus-sensitive and virus-resistant cells were electrophoresed adjacent to each other and to reference DNAs allowed length increases of 20 bp or more to be detected. The equal lengths of product from sensitive (WT) and resistant host strains (V3R, V60R, V65R, etc.) confirm that no enlargement of either CRISPR locus accompanied the acquisition of viral resistance in the strains tested. Length references are provided by BstEII restriction fragments of λ (1.4, 1.3, and 0.7 kbp) and Tsp509I fragments of pUC19 (955 bp, 988 bp).

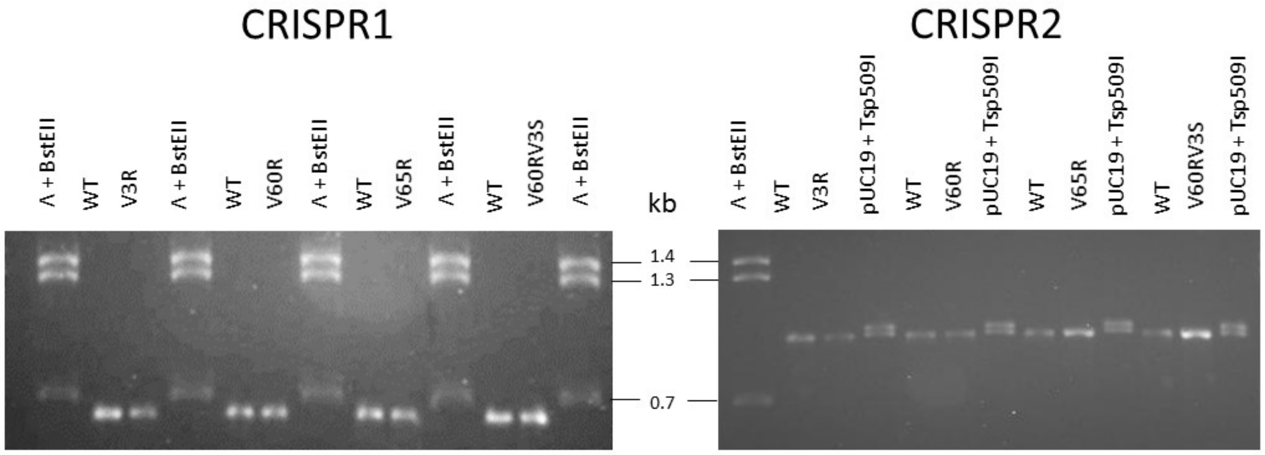

Figure S6. Acquired sensitivity to virus infection.

Scoring of individual colonies from a V3-resistant clone grown to form a population of 10^8 cells (see Materials and Methods). Panels show a set of 48 resuspended colonies spotted on virus-free medium (left) and on medium spread with V3 (right).

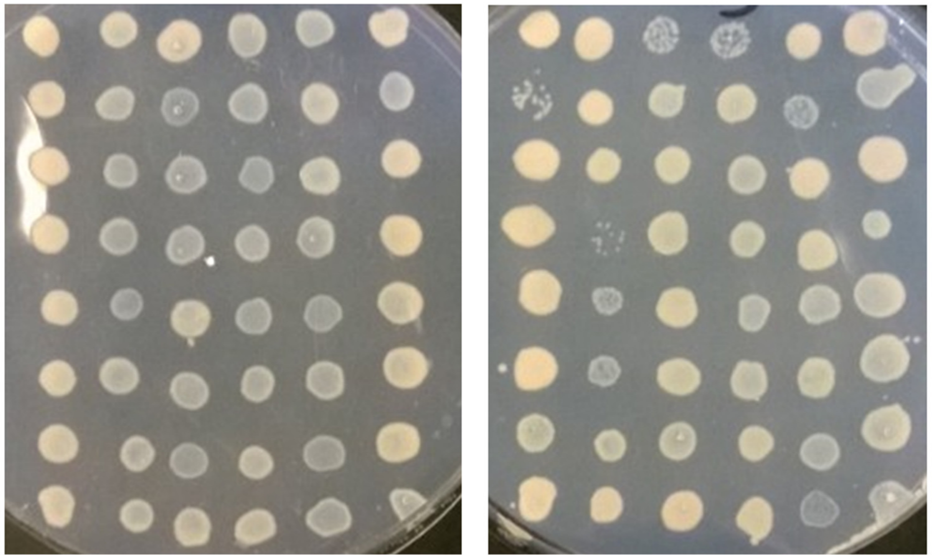

Figure S7. Genetic organization of *S. islandicus pilA1-pilA2* region.

Horizontal bars indicate the DNAs amplified by PCR primers used in this study (Table S1). In host strain K0016-4, the indicated A1 and A2 regions are 895 bp and 868 bp long, respectively, and they occur 2000 bp apart; thus the region depicted below spans M16_4 genome co-ordinates 2551098 to 2554439. Location and orientation of each coding sequence is indicated by the large gray arrows. The open boxes below the DNAs depict the region repeated between the two *pilA* genes, and the vertical bars mark the approximate locations of 26 bp that differ between the two repeats.

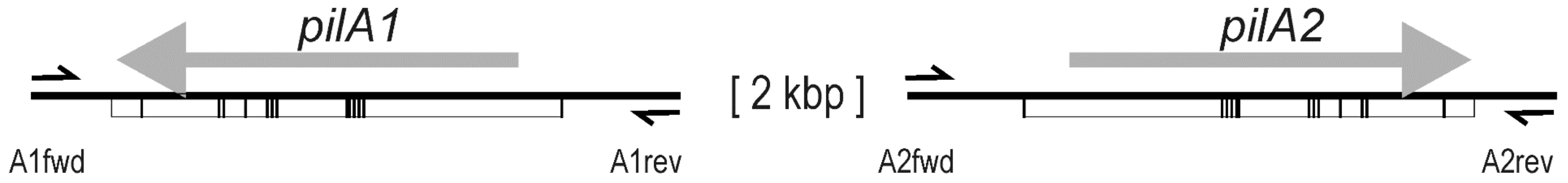

Supplement: Supplementary file 1 [file viruses-14-01439-s001.zip › viruses-1597921-supplementary.pdf]
